# Adapted Taekwondo Training for Prepubertal Children with Developmental Coordination Disorder: A Randomized, Controlled Trial

**DOI:** 10.1038/s41598-018-28738-7

**Published:** 2018-07-09

**Authors:** Ada W. W. Ma, Shirley S. M. Fong, X. Guo, Karen P. Y. Liu, Daniel Y. T. Fong, Young-Hyeon Bae, Lily Yuen, Yoyo T. Y. Cheng, William W. N. Tsang

**Affiliations:** 1 Department of Health and Physical Education, Education University of Hong Kong, Tai Po, Hong Kong China; 2 School of Public Health, University of Hong Kong, Pokfulam, Hong Kong China; 3 Department of Rehabilitation Sciences, Hong Kong Polytechnic University, Hung Hom, Hong Kong China; 4 School of Science and Health (Occupational Therapy), Western Sydney University, Sydney, Australia; 5 School of Nursing, University of Hong Kong, Pokfulam, Hong Kong China; 6 Rehabilitation Clinical Research Center, Korea Workers’ Compensation and Welfare Service, Daegu Hospital, Daegu, Republic of Korea; 7 Heep Hong Society, Kowloon, Hong Kong China

## Abstract

This study evaluated the effectiveness of adapted Taekwondo (TKD) training on skeletal development and motor performance in children with developmental coordination disorder (DCD). One hundred forty-five prepubertal children with DCD were allocated to either the TKD or control groups. Children in the TKD group participated in a weekly 1-hour adapted TKD intervention and daily TKD home exercises for 12 weeks. The primary outcome (delay in skeletal development) and secondary outcomes (Movement Assessment Battery for Children (MABC) total impairment score, eye–hand coordination (EHC) scores, and a standing balance score) were measured at baseline, after the intervention and 3 months after the intervention. Skeletal development improved in both groups over time (*p* < 0.017). The TKD group had a significant delay in skeletal development at baseline compared to the control group (*p* = 0.003) but caught up with the controls at 3 months (*p* = 0.041). Im*p*rovements in the MABC scores were also seen in both groups across time (*p* < 0.017). Only the TKD group had a significant improvement in the EHC movement time at 3 (*p* = 0.009) and 6 months (*p* = 0.016). The adapted TKD intervention may be effective in improving the skeletal development and EHC movement time of children with DCD. For motor performance, the effect of maturation might be more profound.

## Introduction

Developmental coordination disorder (DCD) is one of the most common motor-based disorders and affects approximately 6% of children studying in primary schools^1^. Children with DCD are characterized by their marked motor impairments, such as poor body balance^2–6^ and eye–hand coordination (EHC)^7^. These deficits in upper and lower limb movement are detrimental to the development of overall motor skills and limit participation in activities^3,4^. In particular, poor body balance, which affects 73% to 87% of children with DCD, may result in a predisposition to falls and injuries in daily life^4^, which is a major concern of most parents. Active alternative treatment strategies are required to remediate the motor and balance impairments in this group of children.

In addition to motor impediment, our research team found that skeletal development is also compromised in children with DCD, which is associated with the sedentary lifestyle of these children^8^. The delay in skeletal development, which is reflected by bone age, is also associated with lower bone mineral density^9^ and bone mineral mass in skeletally immature individuals^10^. Therefore, the establishment of an active treatment program to enhance skeletal development and bone health in children with DCD is essential.

Sports training is often an enjoyable and practical way to improve both motor skills and skeletal development in young people. For example, training in taekwondo (TKD, a Korean martial art and a popular combat sport among children and adolescent worldwide) has been shown to be effective in improving bone health in young people^11,12^. Shin *et al*.^11^ reported that the bone mineral density of the lumbar spine was higher in growing, adolescent TKD athletes than in sedentary control subjects because TKD is a weight-bearing and contact sport. We hypothesized that TKD training might also facilitate skeletal development in children with DCD.

TKD is also famous with its kicking techniques, in which single-leg stance stability is crucial and is a determining factor of success in competitions^13^. In our previous studies, we found that as little as 3 months of TKD training could improve gross motor proficiency, such as single-leg standing balance control, vestibular function^14^, and knee muscle strength^2^, in children with DCD^2,14^. Although no study has yet specifically investigated the effects of TKD training on the improvement of upper limb motor performance in this group of children, we postulated that TKD exercises might enhance proficiencies such as eye–hand coordination because visual-motor training (e.g., focus mitt training drills) is emphasized in TKD^15^. Indeed, a previous study has reported that karate (a Japanese martial art similar to TKD) athletes had better eye-hand coordination than non-contact sports athletes^16^. Therefore, the aim of this study was to investigate the effectiveness of a novel adapted TKD training regimen on both skeletal development and motor proficiency, including overall motor performance, eye–hand coordination, and standing balance control, in prepubertal children with DCD.

## Results

### Study population

**Figure 1:**
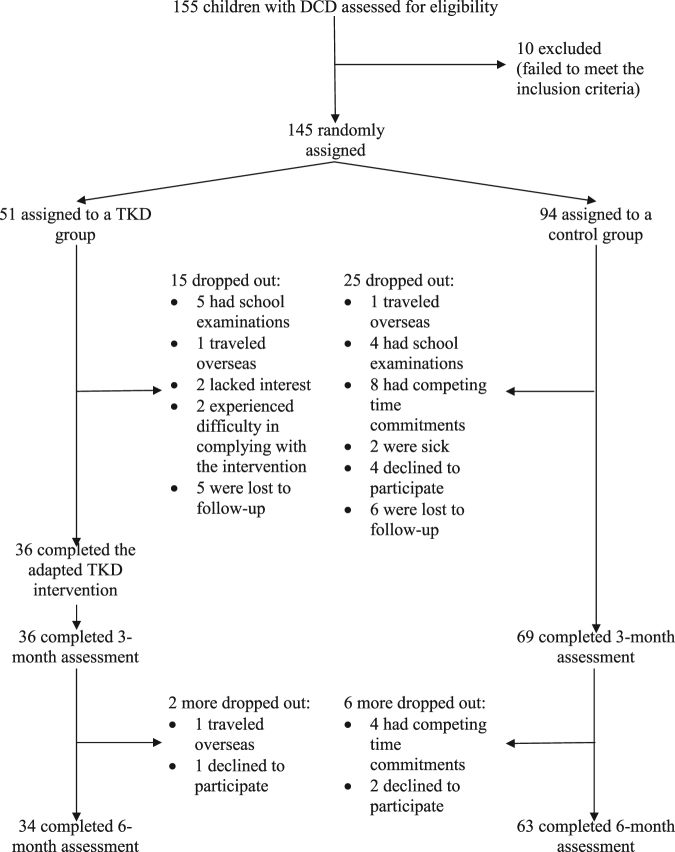
Flow of study.

Between June and October 2016, 155 children with probable DCD were recruited and screened for eligibility. Of the 145 eligible children, 51 were randomly assigned to a TKD group and 94 to a control group. The flow of the participants through each stage of the study is detailed in Fig. 1. Table 1 presents the baseline demographic data of the two groups of participants with DCD. No significant between-groups differences were observed, except that the TKD group had a higher habitual physical activity level than the control group (*p* = 0.002). Baseline physical activity level was therefore treated as a covariate in the statistical analyses. Seventeen participants in the TKD group and 31 participants in the control group dropped out of the trial (Fig. 1). No significant differences were observed between the baseline demographic data of the participants who dropped out and those who completed the study. The average participation rate in the TKD intervention was 75%. All of the participants attended eight sessions (66%) or more. The TKD home exercise compliance rate was 78%. For the control group, the jogging exercise compliance rate was 74% with an average of 9987 steps/day. There were no within-group changes in the participants’ medication used, calcium intake, or sunlight exposure during the study period, and none of the participants received non-study exercise/martial art training.

**Table 1:** Characteristics of DCD participants at baseline.

	TKD group (n = 51)	Control group (n = 94)	*p* value
Age, year	7.4 (1.2)	7.5 (1.2)	0.530
Sex (male/female), n	45/6	76/18	0.263
Weight, kg	24.6 (5.6)	25.8 (7.3)	0.323
Height, cm	124.4 (9.0)	124.4 (9.8)	0.962
Body mass index, kg/m^2^	15.8 (2.0)	16.3 (2.4)	0.177
Arm length, cm	53.2 (4.6)	53.1 (6.4)	0.923
DCD Questionnaire 2007 total score	35.3 (9.0)	37.1 (10.1)	0.301
Calcium intake, mg/day	734.1 (261.8)	754.8 (154.2)	0.689
Time spent in outdoor activities (sunlight exposure), hours/week	4.0 (1.3)	3.9 (0.6)	0.665
Habitual physical activity level, metabolic equivalent hours/week	14.1 (12.3)	7.8 (7.6)	0.002*
Comorbidity, n (%)			0.439
Attention deficit hyperactivity disorder	8 (15.7%)	28 (29.8%)	
Dyslexia	6 (11.8%)	10 (10.6%)	
Autism spectrum disorder	22 (43.1%)	46 (48.9%)	
Medication, n (%)			0.535
Ritalin	5 (9.8%)	7 (7.4%)	
Concerta	1 (2.0%)	4 (4.3%)	
Unknown	2 (3.9%)	7 (7.4%)	

Means (standard deviations) are presented unless otherwise specified.

Abbreviations. DCD = developmental coordination disorder, TKD = Taekwondo.

*p < 0.05.

### Primary outcome

Table 2 shows the mean values of all outcomes at baseline, 3 months, and 6 months. A significant between-groups difference in skeletal development was found at baseline, but not at 3 or 6 months. Therefore, baseline skeletal development data was treated as a covariate in the statistical analysis. Both TKD and control groups demonstrated improvements in skeletal development (i.e., lesser delay in bone age) over time. When comparing to the baseline, the TKD group improved by 0.74 years at 3 months (95% CI, 0.41 to 1.17; *p* < 0.001) and 0.79 years at 6 months (95% CI, 0.48 to 1.27; *p* < 0.001). In the control group, skeletal development improved by 0.55 years (95% CI, 0.29 to 0.80; *p* < 0.001) and 0.68 years (95% CI, 0.47 to 0.89; p < 0.001) at 3 months and 6 months, respectively. A separate statistical analysis was performed after the removal of the dropout cases, and similar results were obtained (data not shown).

**Table 2:** Comparison of outcome measures between TKD group and control group and within individual groups.

	TKD group (n = 51)	Control group (n = 94)	Between-groups difference in mean scores (95% CI)			*p* value		
			TKD group vs. Control group	*p* value	Effect size	Group	Time	Group × Time
**Primary outcome**								
Delay in skeletal development (chronological age – bone age), years						0.990	<0.001*	0.959
Baseline	1.17 (1.76)	0.21 (1.60)	0.96 (0.30, 1.47)	0.003^b^	0.57			
3 months	0.43 (1.61)^a^	−0.34 (1.78)^a^	0.77 (0.03, 1.26)	0.041	0.45			
6 months	0.38 (1.68)^a^	−0.47 (1.72)^a^	0.85 (0.07, 1.31)	0.029	0.50			
**Secondary outcomes**								
MABC total impairment score						0.535	<0.001*	0.134
Baseline	20.53 (7.45)	19.58 (7.70)	0.95 (−1.50, 3.76)	0.396	0.13			
3 months	17.01 (8.08)^a^	17.47 (9.60)^a^	−0.46 (−3.58, 2.73)	0.790	0.05			
6 months	15.74 (8.86)^a^	17.27 (9.88)^a^	−1.53 (−4.28, 2.32)	0.558	0.16			
EHC accuracy value, mm						0.991	0.683	0.911
Baseline	53.41 (77.78)	79.75 (85.75)	−26.34 (−55.97, −0.37)	0.047	0.32			
3 months	28.85 (57.88)	41.35 (68.47)	−12.50 (−34.64, 7.85)	0.214	0.20			
6 months	29.27 (57.75)	39.08 (67.14)	−9.81 (−31.71, 10.37)	0.317	0.16			
EHC reaction time, ms						0.280	0.310	0.706
Baseline	523.09 (153.19)	486.36 (147.09)	36.73 (−14.92, 87.78)	0.163	0.24			
3 months	538.60 (211.02)	497.85 (194.14)	40.75 (−27.87, 109.88)	0.241	0.20			
6 months	541.29 (200.73)	513.91 (224.79)	27.38 (−50.85, 98.69)	0.528	0.13			
EHC movement time, ms						0.507	0.004*	0.390
Baseline	700.42 (242.71)	648.16 (292.26)	52.26 (−47.15, 143.42)	0.320	0.19			
3 months	645.50 (265.01)^a^	624.51 (294.89)	20.99 (−75.81, 120.77)	0.652	0.07			
6 months	644.15 (287.08)^a^	613.66 (291.43)	30.49 (−73.02, 126.74)	0.596	0.11			
mCTSIB composite sway index						0.937	0.092	0.915
Baseline	2.28 (0.71)	2.27 (0.64)	0.01 (−0.22, 0.24)	0.948	0.01			
3 months	2.28 (0.54)	2.23 (0.44)	0.05 (−0.12, 0.21)	0.617	0.10			
6 months	2.11 (0.43)	2.10 (0.38)	0.01 (−0.13, 0.14)	0.962	0.02			

Means (standard deviations) are presented unless otherwise specified.

Abbreviations. MABC = Movement Assessment Battery for Children, EHC = eye–hand coordination, mCTSIB = modified Clinical Test of Sensory Integration of Balance, TKD = Taekwondo, CI = confidence interval.

*p < 0.05.

Within group: ^a^*p* < 0.017 (Bonferroni adjusted) when compared with baseline values.

Between groups: ^b^*p* < 0.017 (Bonferroni adjusted).

### Secondary outcomes

Improvements in the MABC total impairment scores were seen in both the TKD and control groups over time. When comparing to the baseline value, the TKD group’s MABC total impairment score improved by 3.52 points at 3 months (95% CI, 1.85 to 5.42; *p* < 0.001) and 4.79 points at 6 months (95% CI, 2.32 to 6.46; *p* < 0.001). In the control group, the MABC total impairment score improved by 2.11 points (95% CI, 0.94 to 3.22; *p* < 0.001) and 2.31 points (95% CI, 1.20 to 3.37; *p* < 0.001) at 3 months and 6 months, respectively. No significant between-groups differences were found at all time points (Table 2).

Regarding the EHC outcomes, only the TKD group had a significant improvement in movement time at 3 months (54.92 ms compared to the baseline value; 95% CI, 13.04 to 85.57; *p* = 0.009) and 6 months (56.27 ms compared to the baseline value; 95% CI, 10.82 to 100.74; *p* = 0.016). However, the mean differences between the two groups at all time points were not significant. The EHC accuracy value and reaction time remained relatively stable in both groups across time, and none of the between-groups differences were significant. The mCTSIB composite sway index also showed no significant within-group changes or between-groups differences at any time point (Table 2).

### Adverse events

No major adverse events were reported during the intervention or assessments.

## Discussion

This study is the first to show that a 3-month program of adapted TKD training could be effective in improving skeletal development and EHC (in terms of movement time) in children with DCD. Continuous improvement in the EHC outcome was observed for 3 months after the cessation of training. However, adapted TKD training may not be able to improve the overall motor performance or static standing balance performance of children with DCD.

The improvement in skeletal development in the TKD group at 3 months supports our hypothesis that TKD training can facilitate skeletal development in children with DCD. Although the control group also improved over time, which suggests that the effect of maturation cannot be ignored, the improvement was greater in the TKD group (0.74 years) than in the control group (0.55 years). The TKD group had a significant delay in skeletal development at baseline compared to the control group but caught up with the control subjects at 3 months. These findings suggest that TKD training could hasten the development of bone in children with DCD and are consistent with the bone-strengthening benefit of TKD reported in previous studies^11,12^, possibly because TKD training (e.g., striking and blocking maneuvers and jump kicks) places a considerable amount of dynamic, high-magnitude, and high-velocity mechanical stress on growing bones (e.g., the radius and ulna) and cartilage^15^. These external forces may act on the skeleton to stimulate and hasten cartilage growth and chondro-osseous development^17^. However, the mechanisms behind the continuous improvement in skeletal development after the cessation of TKD training remain less understood. The improvement may be primarily due to maturation, as both groups demonstrated similar improvements from 3 to 6 months. Further exploration is warranted to confirm the longer-term effects of adapted TKD training on skeletal development in children with DCD.

Another encouraging finding of this study was that EHC movement time improved from baseline to 3 months and 6 months exclusively in the TKD group. EHC movement time is a measure of the biomechanical delay required to generate sufficient muscle force to complete a finger-pointing task^18^. Thus, our result reveals that training in TKD could probably shorten the biomechanical delay in generating muscle forces in the upper limbs. This finding is consistent with previous studies that suggested that martial arts athletes have better motor control and coordination of the upper limbs, which may be related to the repeated practice of martial art skills^19,20^. Our results further show that this improvement continued until 3 months after the cessation of TKD martial art training, although the underlying mechanisms are still not clear and require further research.

Although EHC movement time improved in the TKD group, EHC reaction time and accuracy did not change over time in either group. A previous study reported that TKD practitioners with more than 3 years of training had faster reaction times than control subjects in response to a visual stimulus^21^. Therefore, we postulate that 3 months of TKD training may not be long enough to elicit a training effect in EHC reaction time. Another plausible reason was that our reaction time test (a finger-pointing task) results could be confounded by the attention level of the participants with DCD who are known to be inattentive to motor tasks^22^. Therefore, further study may monitor the attention level of the participants concurrently during the EHC test^23^. As for the EHC accuracy value, because coordination is task specific, the gross eye–hand coordination skills (e.g., punching a shifting focus mitt) developed through TKD training may not be carried over to the static finger-pointing task, which requires fine motor control^24^.

We found that both TKD and control groups demonstrated similar overall improvements in gross and fine motor performance over time. Thus, the effect of maturation (spontaneous improvement) may be more profound than that of TKD training. With respect to static standing balance performance, surprisingly, neither group showed significant improvement over time. This could be attributable to the fact that testing static balance in a bipedal stance on a force platform was not sufficiently challenging to reflect the participants’ actual balance ability^14^. Further studies may consider using dynamic balance tests such as the Star Excursion Balance Test^25^ to assess the dynamic and functional balance performance of children with DCD.

This study has some limitations. First, due to the nature of TKD intervention, the participants cannot be blinded to group assignment. The optimism of the TKD group participants about the potential benefits of the exercise intervention may have introduced some bias to the results^26^. Second, the findings of this laboratory-based study may not be generalizable to non-laboratory environments such as outdoor or clinical settings. Further studies should examine whether the skeletal and EHC improvements in children with DCD are clinically meaningful and take individual differences into account. Third, the TKD intervention lasted for only 3 months, which may not be long enough to induce marked skeletal development. A future study could include a longer-term TKD intervention and assess the long-term effectiveness of such an intervention. Finally, ultrasonic bone age assessment may not be sensitive enough to detect minimal changes in bone growth. Biomarkers of bone growth could be examined instead.

## Conclusions

The 3-month adapted TKD program may be effective in improving skeletal development and EHC movement time in children with DCD. However, with respect to overall motor performance, the effect of maturation might be more profound than that of TKD. In addition, the TKD intervention may not be able to improve the static standing balance performance, EHC reaction time and finger-pointing accuracy of children with DCD.

## Methods

### Design overview

This prospective, randomized, single-blind and controlled trial was registered at ClinicalTrials.gov (NCT02635711) on December 21, 2015 and was approved by the Human Research Ethics Committee of the University of Hong Kong (reference number: EA1501110). Written informed consent was obtained from each participant and parent, and all procedures were conducted in accordance with the Declaration of Helsinki. Figure 1 depicts a flow diagram of the study.

### Participants

Children with DCD were recruited from nongovernmental organizations, primary schools, and parents’ groups through poster and online advertising and from our research team’s database of DCD study participants. The inclusion criteria were as follows: age between 6 and 9 years; Tanner stage I; diagnosis of DCD based on the Diagnostic and Statistical Manual of Mental Disorders V^1^; a gross motor composite score of 42 or less on the Bruininks-Oseretsky Test of Motor Proficiency^27^ or a total impairment score below the 5th percentile on the Movement Assessment Battery for Children (MABC)^28^; a total score of less than 46 (for children 5 to 7 years 11 months of age) or less than 55 (for children 8 to 9 years 11 months) on the DCD Questionnaire 2007^29^; attendance at a mainstream primary school; normal intelligence; ability to follow instructions; and no experience in TKD or other martial arts. The exclusion criteria were as follows: neurological or other movement disorder; diagnosis of congenital, sensory, musculoskeletal, cardiopulmonary, or psychiatric disorder that may affect skeletal development or motor performance; active treatment such as complementary and alternative medicine that may confound the effects of the intervention; or demonstration of excessive disruptive behavior (as self-discipline is important in a TKD class)^15^.

### Screening and randomization

Two physiotherapists first screened the volunteers by telephone, and those who seemed eligible underwent an in-person evaluation and baseline assessment. All eligible participants were randomly assigned to either a TKD group or a control group. The randomization procedure was performed by an independent person with a computer-generated randomization sequence (www.random.org) and sealed opaque envelopes to ensure concealed allocation.

### Intervention

The participants who were assigned to the TKD group underwent an adapted TKD training regimen (detailed in Table 3) that was developed by our research team^14^ to train balance control and eye–hand coordination and facilitate skeletal development in children with DCD. The training protocol was similar to the traditional TKD training protocol for beginners (offered to the general public of similar age)^15^ except that our participants spent more time on striking and kicking techniques. It is because we postulated that the high-impact striking techniques (e.g., punching and blocking) incorporated in the program might stimulate bone growth^17^, whereas the kicking and striking focus mitt practice might improve balance control^14^ and eye–hand coordination^30^, respectively. The TKD group attended a weekly 1-hour session of adapted TKD training that was held at the University of Hong Kong for 12 weeks. All TKD training sessions were conducted by a World Taekwondo Federation black belt coach and an assistant coach. In addition, each participant was given TKD home exercises to increase the training frequency to 7 times/week^14^. The home exercises were the same as those practiced during the supervised TKD sessions. Parents were required to document the child’s TKD home practice in a logbook. The active control group received no TKD training but jogged for 1 hour per day for 12 weeks in their natural environment. Pedometers were used to monitor the exercise level, and the pedometer counts (steps/day) were documented by the parents in a logbook.

**Table 3:** Adapted taekwondo training protocol for children with DCD.

Exercise/TKD technique	Aims and features	Frequency	Intensity	Duration
Warm up	• Jogging to increase body temperature	TKD class: once/week; home practice: daily (excluding the TKD class days)	Mild sweating	5 min
Stretching	• Static stretch of major muscle groups to improve flexibility		Mild tension of muscles	5 to 10 min
Body punch (with and without a moving focus mitt)	• Striking blow with a closed fist – compressive force is transmitted through the metacarpals, wrist, forearm bones to the upper arm and body• Strike a moving target – requires visual-motor ability, fast reaction, coordinated limb movements and concentration		20 reps, performed with alternate arms. 20 reps for each technique, performed with alternate arms.	5 to 10 min 15 to 20 min
Blocking techniques: Rising block (in form of set-sparring)	• Block a punch/attack with forearm – ulna and/or radius are subjected to shear load and compressive load due to strong contraction of forearm muscles			
• Outside/side block (in form of set-sparring)				
• Inside block (in form of set-sparring)				
• Down block (in form of set-sparring)				
Kicking techniques: Front kick in fighting stance (with and without a kick pad)	• Require postural adjustment, proper body alignment and standing balance.		40 reps for each technique, performed with alternate legs.	15 to 20 min
• Round house kick in fighting stance (with and without a kick pad)				
• Side kick in fighting stance (with and without a kick pad)				
• Back kick in fighting stance (with and without a kick pad)				
Cool-down and stretching	• Walking to lower body temperature• Static stretch of major muscle groups to improve flexibility			5 min

### Test procedures and blinding

All the participants were assessed before the start of the intervention (baseline), shortly after the 3-month intervention (post-test), and 3 months after its completion (follow-up test) by two trained research assistants who were blinded to the intervention allocation. Data collection took place in the Physical Activity Laboratory at the University of Hong Kong.

### Demographics

Age, sex, body weight, height, arm length, hand dominance, indoor and outdoor exercise habits, comorbid conditions, medical history, and treatments received were obtained from medical records, if available, and by interviewing the participants and parents. Body mass index was calculated by dividing body weight (kg) by the square of height (m). Habitual physical activity level was calculated based on the exercise intensity, duration, frequency, and the assigned metabolic equivalent (MET) value of the physical activity according to the Compendium of Energy Expenditures for Youth^31^. Dietary calcium intake was estimated using the Hong Kong Hospital Authority Hong Kong East Cluster’s Nutrition Information website (http://www3.ha.org.hk/dic/nq_03.html).

### Primary outcome

The skeletal development of each participant was determined by ultrasonography using the Sunlight BonAge system (Sunlight Medical Ltd., Tel Aviv, Israel), which provides an accurate assessment of skeletal age in children (intra-operator precision: 0.24 years for boys and 0.25 years for girls). The results obtained from this ultrasonic bone age measurement are highly correlated with the conventional Greulich and Pyle method^32,33^. During the test, each participant rested his or her left forearm on the measurement table of the BoneAge device. The assessor helped the participant to position and stabilize the distal tip of the left ulna styloid process between the two ultrasound transducers of the device. Ultrasonic waves at 750 kHz were then transmitted through the left wrist between the two ultrasound transducers. A total of 5 to 11 cycles of measurement were performed to ensure high precision. The BonAge device calculated the speed of sound and uses the distance between the two transducers, under known and controlled pressure conditions, and a proprietary sex- and ethnicity-based algorithm to provide a numeric result of bone age^32,33^. The ‘delay in skeletal development’ was then calculated by subtracting the bone age from the participant’s chronological age^8^.

### Secondary outcomes

The MABC was used to assess the motor proficiency of all participants. It is a standardized, validated and reliable tool for measuring gross and fine motor performance in children between 4 and 12 years of age^28,34^. The assessment comprises eight tasks for each of the four age bands (4 to 6 years, 7 to 8 years, 9 to 10 years, and 11 to 12 years). The eight tasks are divided into three domains: manual dexterity, ball skill, and static and dynamic balance. The detailed assessment items are described in Henderson and Sugden^28^. Each participant was assessed with the appropriate age-band tests according to their chronological age. The raw score for each test item was summed to obtain a total impairment score, which was used for analysis. A lower total impairment score represents better motor performance^28^.

Upper limb motor proficiency was assessed with a computerized eye–hand coordination test. The detailed assessment procedures are described in our previous study^35^. In brief, the participant’s dominant hand was placed on a force-sensing resistor (FSR 406, Interlink Electronics, CA) positioned 10 cm from a touch screen (Clear Tek 3000 LCD screen, MicroTouch Systems Inc., Methuen, USA). Visual targets in the form of a ball (1.2 cm in diameter) appeared on the screen in a random order. The participant was instructed to touch each target with the index finger of the dominant hand as quickly and as accurately as possible. Each participant repeated this finger-pointing task 15 times. Accuracy (in mm) was defined as the absolute value of the deviation of the participant’s touch location from the center of the visual target^36^. The accuracy scores for each trial were recorded, and the mean accuracy score of 15 trials was used for analysis. A smaller value indicates greater accuracy. In addition, the average reaction time (i.e., the time between the appearance of the visual target on the screen and the moment the thumb leaves the force-sensing resistor) and the average movement time (i.e., the time between the moment the thumb leaves the pressure sensor and that when the index finger touches the ball on the screen) of the 15 trials were calculated. The test–retest reliability of this eye–hand coordination test was found to be moderate (ICC, 0.68 to 0.71)^35^.

Standing balance control was assessed using a modified Clinical Test of Sensory Integration and Balance (mCTSIB), which is one of the most widely used tests of balance performance in children with disabilities^37^. During the test, each participant stood barefoot on the force platform of a Biosway computerized dynamic posturography machine (Biodex Medical Systems Inc., Shirley, NY) and was exposed to four sensory conditions in order: condition 1 – eyes open and firm surface; condition 2 – eyes closed and firm surface; condition 3 – eyes open and foam surface; and condition 4 – eyes closed and foam surface. The computerized dynamic posturography machine tracked the participant’s sway angle and direction from center over the four sensory conditions and automatically generated a sway index for each condition and a composite sway index (i.e., the average value of the four condition-specific sway indices). The composite sway index, which reflects the participant’s overall static standing balance ability, was used for analysis. A smaller sway index indicates better balance performance^38^.

### Statistical analyses

Sample size was calculated using G*Power version 3.1.0 (Franz Faul, Universitat Kiet, Germany). Based on our pilot study, an average effect size of 0.99 was assumed for the primary outcome measure. Because a dropout rate of 25% was anticipated^14^, with 80% power and an alpha level of 5% (two-tailed), a minimum of 23 participants per group was required.

Statistical analyses were performed with SPSS 24.0 (IBM, Armonk, NY) and based on an intention-to-treat (last-observation-carried-forward) assumption. Descriptive statistics were produced for all of the variables. The normality of the data was checked using the Kolmogorov-Simirnov test and/or histograms. Between-groups differences in demographic and baseline outcome variables were tested with an independent *t*-test for continuous data and a chi-square test for categorical data. The effects of TKD on primary and secondary outcomes were compared by means of mixed-model repeated-measures analysis of covariance with adjustment for demographic (habitual physical activity level) and baseline covariates. The between-subjects factor was group, and the within-subject factor was time. Post hoc tests were conducted only if the results of the analysis of covariance indicated that the null hypothesis should be rejected. Independent *t*-tests with 95% confidence intervals (CIs) were used to compare group means, and paired *t*-tests were used to examine the within-group changes from baseline to 6 months. All *p* values were subjected to Bonferonni adjustment to maintain an overall two-tailed significance level at 5%.
